# Comparison of MDCT Imaging and HR-pQCT for Assessment of Osseus Consolidation in Symptomatic Scaphoid Non-Union Treated with Avascular Bone Grafting and Percutaneous Screw Fixation—A Prospective Clinical Pilot Study

**DOI:** 10.3390/jcm14051476

**Published:** 2025-02-22

**Authors:** Irena Krusche-Mandl, Sabrina Holzer, Kevin Döring, Arastoo Nia, Géraldine Désirée Sturz, Maximilian F. Kasparek, Janina M. Patsch, Iris-Melanie Noebauer-Huhmann, Jochen Erhart, Stefan Hajdu

**Affiliations:** 1 Department of Orthopedics and Trauma Surgery, General Hospital of Vienna, Medical University of Vienna, Währinger Gürtel 18-20, 1090 Vienna, Austria; irena.krusche-mandl@meduniwien.ac.at (I.K.-M.); kevin.doering@meduniwien.ac.at (K.D.); geraldine.sturz@gmail.com (G.D.S.); max.kasparek@hotmail.com (M.F.K.); jochen.erhart@bbeisen.at (J.E.); stefan.hajdu@meduniwien.ac.at (S.H.); 2 Department of Orthopedics and Trauma Surgery, Krankenhaus der Barmherzigen Brüder Eisenstadt, 7000 Eisenstadt, Austria; 3 Department of Orthopaedic Surgery and Trauma Surgery, Evangelisches Krankenhaus, 1180 Vienna, Austria; 4 Department of Biomedical Imaging and Image-Guided Therapy, General Hospital of Vienna, Medical University of Vienna, 1090 Vienna, Austria; janina.patsch@meduniwien.ac.at (J.M.P.); iris.noebauer@meduniwien.ac.at (I.-M.N.-H.)

**Keywords:** scaphoid non-union, HR-pQCT, MDCT, avascular iliac grafting, scaphoid non-union surgery

## Abstract

**Background/objectives:** This study prospectively evaluated clinical outcomes and osseous consolidation in patients with symptomatic scaphoid non-union treated with avascular bone grafting and percutaneous screw fixation. Two imaging methods, MDCT (multi-detector computed tomography) and HR-pQCT (high-resolution peripheric quantitative computer tomography), were employed to assess bone healing. **Methods:** In Vienna, eight consecutive patients with nine symptomatic scaphoid non-unions underwent revision surgery. Clinical outcomes were measured using DASH and PRWE scores, grip strength, and thumb strength. MDCT and HR-pQCT imaging were conducted 6 and 12 weeks post-operatively. **Results:** The median DASH score improved significantly from 43.3 (range 3.3–76.7) pre-operatively to 26.6 (*p* = 0.024) at 3 months and 16.2 (*p* = 0.06) at 12 months post-operatively. At 5–6 years, the median DASH score was 2 (range 0–15). At 6 weeks, both MDCT and HR-pQCT detected >50% bone healing at the distal interface. At the proximal interface, HR-pQCT detected >50% healing in all cases, whereas MDCT still showed <50% healing in 25% of cases. By 12 weeks, both methods demonstrated >50% osseous consolidation at both interfaces. **Conclusions:** Avascular iliac grafting with screw fixation achieved excellent long-term clinical outcomes for symptomatic scaphoid non-union. HR-pQCT proved superior to MDCT for assessing early bone healing.

## 1. Introduction

The adequate clinical and radiological diagnosis of scaphoid fractures still remains a challenge and is often overlooked in daily clinical practice [1,2]. Therefore, scaphoid non-union is still a common pathology with functional impairment and pain for patients [3,4]. Avascular bone grafting and screw fixation is a successful procedure in the surgical treatment of symptomatic scaphoid waist fracture non-union, with reported healing rates of 50–94% [4,5,6] and an average healing rate of 88%, as reported in a meta-analysis led by Janowski et al. [3]. Prognostic consolidation factors include time between fracture and surgery, fracture localization, instability, and presence of avascular necrosis [3,4,7,8,9]. Thus, a delayed time to surgery correlates with a significant drop in healing rates of below 80% if 12 months are exceeded [4]. There is a lack of consensus regarding the optimal duration of post-operative immobilization following revision surgery, but the majority of authors advocate a post-operative immobilization time ranging from 4 to 12 weeks [10,11,12,13] until radiographical or MDCT evidence of trabecular healing has occurred. Healing criteria as established by Fernandez include the absence of pain, radiographic evidence of bridging bony trabeculae across both sides of the interposed graft, the disappearance of interface lines on conventional X-rays, and the absence of signs of screw loosening [14].

For post-operative evaluation, X-ray and MDCT are used. X-rays provide two-dimensional images; however, they are limited in providing detailed analysis of fracture healing, often underestimating scaphoid union [4]. MDCT imaging provides a three-dimensional evaluation; however, only the macrostructure of the cortical bone can be analyzed with the disadvantage of radiation exposure [15].

The High-resolution peripheral quantitative CT (HR-pQCT) has been successfully used in bone mineral research, when measuring bone mineral density, fracture detection and the monitoring inflammatory joint disorders or anti-osteoporotic therapies (van den Bergh). It is a noninvasive technique that provides three-dimensional images of peripheral bones with a low radiation dose and a higher spatial resolution compared to MDCT imaging techniques [16,17]. Current studies estimate that more than one-third of scaphoid fractures are missed by current application of MDCT scanning in clinically suspected scaphoid fractures compared to HR-pQCT, mainly due to better visualization of the microarchitectural bone structure [18,19,20,21].

In a prior study, the healing process of distal radius fractures under conservative treatment with HR-pQCT was evaluated [21,22]. Twelve weeks post-fracture, the main changes seen were related to trabecular thickness as well as bone stiffness, yet no significant changes in trabecular density were observed [21].

To date, only two studies have employed HR-pQCT to assess bone healing in scaphoid fractures. The first, published in 2021, involved a cohort of eleven patients with a follow-up period of 26 weeks, reporting that fracture healing had not yet been completed at this time [12]. The second, published in March 2025, focused on the detailed bone remodeling of conservatively treated acute scaphoid fractures. This study demonstrated complete bone consolidation in half of the fractures at 12 weeks and in all fractures at 52 weeks post-trauma, with no cases of non-union. Furthermore, the authors concluded that HR-pQCT exhibited superior sensitivity compared to multi-detector computed tomography (MDCT) in detecting full bone consolidation following scaphoid fractures [23].

To our knowledge, no studies have employed HR-pQCT to assess partial consolidation (i.e., 50%) or explored its use in the post-operative evaluation of consolidation in scaphoid non-union.

Still, MDCT scanning after surgical treatment with implants is challenging due to metal artifacts [24].

The aim of the current study was to evaluate osseus consolidation using X-ray, MDCT imaging, and HR-pQCT to determine the best imaging technique for the analysis of osseus consolidation in avascular bone grafting and screw fixation in symptomatic scaphoid non-unions. Additionally, clinical and functional results were evaluated prospectively.

## 2. Materials and Methods

The current prospective study analyzes eight consecutive patients who underwent surgery due to nine symptomatic scaphoid non-unions between February 2011 and October 2013. All patients underwent avascular bone grafting with screw fixation. Post-operative wrist immobilization of at least 8 weeks was applied. This study was approved by the institutional review board and written informed consent was obtained from each patient. We included all consecutive skeletally mature patients who were treated for symptomatic scaphoid non-union in all thirds of the bone with avascular bone grafting and screw fixation in our Level I Trauma Center and had a minimum follow-up of 3 months. Concomitant injuries which seemed irrelevant to the healing potential of the scaphoid non-union were not excluded; we had one case of bony avulsion of the triquetrum, with one forearm shaft fracture, one distal radial fracture, and fracture of the hamate in one patient. We excluded any patients with avascular necrosis or osteoarthritis (SNAC: scaphoid non-union advanced collapse) and all patients who were not willing or able to follow protocol or would not have given their written consent. Further patients who were pregnant or had tremors were excluded.

All patients were male and had a mean age of 28 years (range 16–42 years) at the time of surgery. The dominant hand was involved in six cases and the non-dominant hand was involved in three cases. In one patient, there was a bilateral scaphoid non-union. Overall demographic data and fracture characterization are displayed in Table 1.

All patients had a history of trauma. One patient underwent screw osteosynthesis initially, eight patients (nine with scaphoid non-union, one patient with bilateral injury) underwent conservative treatment primarily, and one did not have any initial treatment. Three patients had accompanying injuries. One patient had a forearm fracture, one patient had a bony avulsion of the triquetral bone, and one patient had a fracture of the distal radius, including a fracture of the volar rim, as well as an intermediate fragment and a fracture of the hamate bone. An average of 30 months was observed between trauma and surgery (range 8–123).

None of the included patients had any medical history like diabetes, coronary heart disease, high blood pressure, rheumatic diseases, therapy with corticosteroids, malignant tumor, stroke, asthma, chronic obstructive pulmonary disease, or osteoporosis. None of the patients had had carpal tunnel syndrome prior to surgery. Two of the included patients were smokers (both 8 pack-years) and five were drinking alcohol on a regular basis. Data on age, affected side, gender, grip and thumb strength, and PRWE and DASH test results were recorded.

### 2.1. Surgical Technique

In all patients, open-reduction, non-vascularized iliac crest graft interposition and internal screw fixation were performed as previously described by Fisk-Fernandez [14]. Therefore, all surgeries were performed using a volar approach. For deeper exposure and better view of the non-union site, the flexor carpi radialis (FCR) was retracted and the palmar wrist capsule was opened and winded, while fibrous tissue was resected at the non-union site and the fractured surfaces were freshened until vascularized bone was revealed, using Kirschner wires as joysticks and spinal retractors to achieve the correct position. In terms of DISI position, the dorsal lunate rotation was corrected by inserting an additional K-wire in the lunate and pinning it temporarily to the radius, the so-called Linscheid maneuver. The pre-shaped tricortical corticocancellous bone graft from the iliac crest was inserted by press-fit. The reconstructed scaphoid was then fixated with a K-wire from the Acutrak^®^ set (Acumed©, Hillsboro, OR, USA) in a central position. A second K-wire was inserted parallel in order to maintain rotational stability. Subsequently, an Acutrak^®^ screw was inserted percutaneously, depending on the non-union site, via a volar or dorsal approach.

### 2.2. Clinical and Radiological Assessment

To assess functional outcome, grip strength, thumb strength, Patient-Rated Wrist Evaluation (PRWE) Score, and the Disability of the Arm, Shoulder and Hand (DASH) Score/questionnaire were assessed at each follow-up visit [26,27]. Clinical data were collected pre-operatively two, four, and six weeks and three, six, and twelve months after surgery. All patients had radiographic examinations that included the scaphoid series (posteroanterior, lateral, oblique, and posteroanterior ulnar deviation view). Moreover, all patients underwent an MDCT and HR-pQCT scan after 6 weeks and 12 weeks to evaluate osseus healing. Radiographic union was defined as bridging trabeculae across the interfaces over 75%.

To quantify a mean percentage of union at each interface, the method described by Singh et al. was used: The total length of trabecular bridging along each interface was measured and divided by the total width of the scaphoid, therefore giving a percentage of union [28]. Pre- and post-operative radiographic evaluation were performed by two experienced observers independently and were assigned to the following groups according to Grewal et al. [29]: 1. non-union (no trabecular structure at the non-union site), 2. tenuously united (below 50% trabecular structure at the non-union site), 3. partially united (50–75% of trabecular structure at the non-union site), and 4. healed (75–100% of trabecular structure at the non-union site).

#### 2.2.1. MDCT Imaging

MDCT scans were obtained after 6 and 12 weeks using a CT scanner (64 slice, Siemens, Munich, Germany) from the distal radioulnar joint to the proximal third of metacarpal bones. We used a bone reconstruction filter and high-resolution reconstruction with 0.5 mm slice thickness. Acquired volumetric data were reconstructed along the longitudinal axis of the scaphoid and axial, and coronal and sagittal scans of the scaphoid could be obtained.

#### 2.2.2. HR-pQCT Imaging

HR-pQCT (HR-pQCT I, Xtreme Ct, Scanco Medical AG, Wangen-Brüttisellen, Switzerland) scans were performed after 6 and 12 weeks post-operatively with the following settings: 70 kVp tube voltage, 114 µA tube current, 300 ms integration time, and 18 µm voxel size. For the scaphoid, a reference line was placed at the proximal edge of the scaphoid. From there, an area of 400 mm in distal direction (total scan area of 400 mm, 350 slices) was scanned in 3 stacks.

During scanning, the wrist was immobilized with a synthetic non-fiberglass cast and standard motion restraining holders for HR-pQCT scanning were used [30]. This was repeated 3 times per scaphoid until the distal edge was reached [31,32]. A calibration phantom was scanned daily to accurately report hydroxyapatite bone mineral densities. Radiation exposure during an HR-pQCT for an average scanning time of 3 min for scaphoid imaging resulted in an effective dose of approximately 15 μSv per scan [32].

#### 2.2.3. Scan Quality Assessment

Scan quality was graded using the system by Pialat et al. to grade HR-pQCT scans of the radius and tibia [33]. A single low-resolution slice of each stack of the scans was graded by the operator during scan acquisition (standard grading). Scans were completely repeated once the quality of at least 1 stack had a grade >3. The scan with the best quality in the 3 stacks was included for further analyses.

#### 2.2.4. Scaphoid Osseous Consolidation Assessment

All HR-pQCT and MDCT scans were independently evaluated by an experienced musculoskeletal radiologist (J.P.) and an orthopedic trauma surgeon (I.K-M) specialized in hand surgery to assess bone consolidation. HR-pQCT scans were exported in Digital Imaging and Communications in Medicine (DICOM) format, and wrist images were reconstructed in transverse, coronal, and sagittal planes. All images and reconstructions were anonymized and uploaded to a local workstation for analysis.

**Figure 1:**
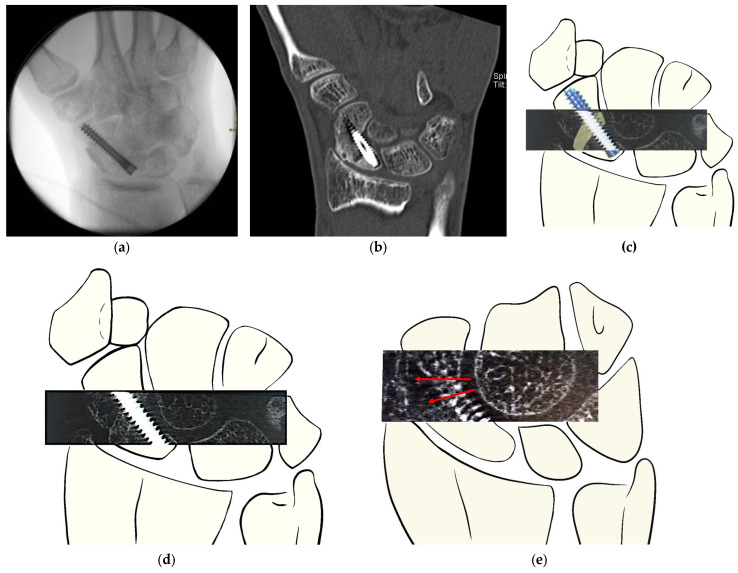
Assessment of bone consolidation after revision surgery of scaphoid non-union with avascular iliac crest graft and percutaneous headless screw fixation. (**a**) Intraoperative imaging after iliac grafting and screw placement. (**b**) Post-operative MDCT scan at 6 weeks showing nearly complete healing at the distal interface and almost no bone consolidation at the proximal interface. (**c**) Schematic illustration of the HR-pQCT stack displaying the iliac graft positioning withing the scaphoid by S. Holzer. (**d**,**e**) Examples of obtained HR-pQCT scans of the same patient at 6 weeks after surgery, demonstrating trabecular structure present in the proximal and distal interfaces. (**d**) Display of well-distinguished ridges of the iliac graft, with clear trabecular structures, yet lacking bony bridging at the interfaces, suggesting resorption at the graft ridge. (**e**) Cross-section at a different scaphoid level; red arrow: display of blurry trabecular formation as described by Bevers et al. [22], a precursor to well-defined trabecular arches (schematic illustration by S. Holzer).

The two observers independently assessed bone consolidation at both the distal and proximal interfaces. For both HR-pQCT and MDCT, evaluations focused on the interface between the distal scaphoid pole and the iliac crest graft, as well as the interface between the graft and the proximal scaphoid pole. An example of the assessment method is illustrated in Figure 1.

### 2.3. Statistical Analysis

Statistical analyses focused on bony incorporation of the distal and proximal interface verified by MDCT and on functional outcomes using the DASH score. Descriptive statistics were performed to analyze medians, frequencies, and ranges of relevant demographic and surgery-specific parameters. Paired *t*-tests were performed to detect differences in post-operative DASH-score. The follow-up was calculated from the date of surgery to the last follow-up visit. A *p*-value of <0.05 was considered significant.

The quantitative measures obtained were statistically analyzed using IBM SPSS Statistics (Version 26.0. IBM Corp, Armonk, NY, USA).

## 3. Results

At 6 weeks, bone healing over 50% was observed at the distal interface in all cases using both scanning methods (MDCT and HR-pQCT) (Table 2). At the proximal site using HR-pQCT imaging, bone healing of more than 50% was recorded in all cases. In comparison to MDCT imaging, below 50% healing was still present in 25%.

After 12 weeks, HR-pQCT imaging as well as conventional CT imaging revealed osseous consolidation of over 50% in all cases at the proximal as well as at the distal interface. MDCT imaging showed higher rates of complete bone healing at the distal interface compared to the HR-pQCT (87.5% and 75%, respectively). In contrast, complete osseous healing was found in MDCT imaging at the proximal interface in 37,5% compared to 50% in the HR-pQCT (Table 3).

In the visual assessment of all scans at 6 weeks in >50% of cases, blurry trabecular formation was observed at the proximal as well as at the distal interface.

Average thumb and grip strength is displayed in Table 4 and Table 5. After an initial drop in strength 6 weeks after surgery, strength recovery to baseline data could already be noted as early as 3 months after surgery, while continuing to increase to similar levels as the contralateral side after 6 months.

The DASH score at baseline averaged a median of 43.3 (range 3.3–76.7) and was 45.8 (range 30.8–70.8) at 6 weeks, it decreased 3 months post-operatively to 26.7 (range 35.6–58.3, *p* = 0.024), and it showed a tendency for improvement to 16.2 (range 4–25.8, *p* = 0.06) at 12 months. After 5 to 6 years, the DASH score median was 2 (range 0–15) (Table 6).

The average of the PRWE score at baseline was 42.5 (range 15–82) and primarily increased to 46.50 (range 31–80) at 6 weeks. After 3 months, it decreased to 40.63 (range 18–65) and to 31.5 (range 14–65) after one year. After 5 to 6 years, the PRWE score showed a mean of 20.38 (range 6 to 43) (Table 7).

## 4. Discussion

The objective of the present study was to prospectively assess the clinical and functional outcomes of avascular bone grafting combined with screw fixation in patients with symptomatic scaphoid non-union. Bone healing was evaluated using MDCT and HR-pQCT imaging to identify the most effective imaging technique for analyzing bone consolidation.

### 4.1. Osseous Consolidation

When considering the overall consolidation rates, a comparison with the literature is difficult, as some authors consider consolidation rates at different endpoints. The duration of immobilization and the follow-up time after non-union are not always mentioned in detail. Moreover, the considered surgical technique differs among published papers [4,34,35,36]. Grewal et al. define scaphoid consolidation as the presence of healing in over 50% of the fracture gap and refer to Scaphoid fracture as being healed when trabecular tissue is present in 75–100% of the fracture gap [29]. Based on this definition, we could confirm a 100% consolidation rate within 12 weeks after surgery using both scanning methods.

At the last follow-up, we could classify 75% of all scaphoids as healed at the distal interface using the HR-pQCT and report a healing rate of at least 50% on the proximal interface. By MDCT, we could classify 87.5% of scaphoids as healed distally and at least 37.5% of all cases as healed at the proximal interface. These numbers confirm our calculated healing estimate by Ramamurthy et al. [25] of 78.4% (range 33–95%) and align with published numbers in the common literature ranging from 76 to 100% [4,34,35,36].

### 4.2. Imaging Techniques in Scaphoid Healing

Osseus consolidation was evaluated with MDCT imaging and HR-pQCT in order to determine the best imaging technique for analysis of osseus consolidation. Bone consolidation of at least 50% was shown in the HR-pQCT scan in the proximal interface in 75% of cases and distally in all cases at an early stage, such as 6 weeks. In contrast, the MDCT showed tenuous consolidation of the proximal interface at six weeks in 25% of cases. We could affirm early detection of trabecular structure and thus bone healing using HR-pQCT at the proximal interface after non-union surgery. A similar observation was also described by Nishino et al. while observing distal radial fracture healing using the HR-pQCT [23].

After an initial resorption process with increased fracture visibility at 3 weeks in HR-pQCT scans, Bevers et al. observed a ‘blurring’ of trabecular structures within the fracture line at 6 weeks, subsequently becoming individual trabeculae which were hardly indistinguishable from neighboring trabeculae [22]. Similar effects could be observed at proximal and distal interfaces while evaluating scaphoid non-unions with the same scanning method.

On the other hand, at 12 weeks, the HR-pQCT scan showed a consolidation of at least 50% at the proximal and distal interface in all cases. Using MDCT, only 62.5% of all cases could be considered as “partially united” at the proximal interface, indicating a higher sensitivity of HR-pQCT to microarchitectural changes. So far, this discrepancy between results in healing estimation according to the scanning technique for scaphoid non-union surgery assessment has not been described in the literature but may reflect HR-pQCT’s superior spatial resolution, allowing for enhanced visualization of microarchitectural changes, which are critical in assessing the complex consolidation processes at the proximal scaphoid.

Our findings suggest that by 12 weeks, bone consolidation after scaphoid revision surgery is achieved in only 50% of cases, highlighting that scaphoid healing is a slow process even after cast removal. Activities of daily living and weight-bearing functions may not fully recover simultaneously, supporting the clinical recommendation to gradually increase wrist loading as pain permits after cast removal. This approach is consistent with previous studies on distal radius fractures and with the recommendations by Bevers et al. Accordingly, significant improvements in DASH score and improved PRWE scores, as well as a noted increase in grip strength, were observed between 3 and 6 months post-operatively. These findings suggest ongoing osseous consolidation and progression of the healing process [22,23].

As previously shown, HR-pCT scan seems to combine the advantage of a more critical view of bone healing behavior whilst carrying the advantage of low radiation doses, with 15 μSv per scan [32]. In relation, background radiation is estimated to be around 4.5 mSv by Austrian agencies, and to be orders of magnitude higher [37].

### 4.3. Limitations and Future Implications

Despite its strengths, this study has several limitations, including a small sample size and a relatively homogenous demographic consisting of male patients only, which limits the generalizability of our findings to a broader population. Nonetheless, this study provides useful preliminary results.

Based on these findings, future research could explore the potential clinical application of HR-pQCT, particularly as an alternative to conventional CT, for determining fracture consolidation or assessing partial consolidation in both scaphoid non-unions and acute fractures. Furthermore, HR-pQCT may be valuable in the post-operative evaluation of surgically managed acute scaphoid fractures.

Incorporating HR-pQCT into clinical practice could facilitate more individualized decision-making regarding the timing of functional treatment initiation and cast removal. Due to its low radiation dose, HR-pQCT imaging could be repeated more frequently, for instance on a two-weekly basis, to minimize immobilization duration and potentially enhance early functional outcomes. Additionally, HR-pQCT could be employed to monitor graft healing behavior over time.

Guss et al. demonstrated in their biomechanical cadaver study that patients with surgically treated scaphoid waist fractures, utilizing a compression screw, may potentially return to unrestricted activity with 50% partial healing from a biomechanical standpoint [38]. To assess the biomechanical relevance of HR-pQCT, comparable biomechanical testing would be required to validate its applicability in predicting functional outcomes and determining the threshold for full recovery.

Finally, expanding the study to include a larger and more diverse patient population would strengthen the evidence supporting the use of HR-pQCT in the management of scaphoid non-unions.

## 5. Conclusions

Surgical treatment with avascular iliac grafting and screw fixation results in excellent clinical outcomes in patients with symptomatic scaphoid non-union. HR-pQCT sensitivity to microarchitectural changes is important in MDCT imaging for the assessment of post-operative bone consolidation, especially in the early detection of trabecular presence. Furthermore, HR-pQCT offers the advantage of lower radiation exposure compared to MDCT, making it a safer imaging option for patients. HR-pQCT is a valuable supplementary imaging modality, enhancing the comprehensive evaluation of scaphoid non-union healing.

## Figures and Tables

**Table 1:** Demographic data and fracture characterization.

Demographic Data	Average (Range)		
Age	Ø 28.4 years (range: 15–42 years)		
Gender (male/female)	8/0		
Dominant hand	5 (62.5%)		
Time from trauma to surgery in months	Ø 30 months (range: 8–123 months)		
**Fracture Charaterization**			
Classification according to Herbert at time of trauma	B2: 2	B3:2	B4:4
Non-union site localization	Proximal third: 60%	Middle third: 21.2%	Distal third: 18.8%
Pseudarthrosis stadium according to Lanz classification	IIb:8		
Healing probability according to Ramamurthy et al. [25] in %	78.4% (33–95%)		
Russe classification	Horizontal oblique 25%	Transverse 37.5%	Vertical oblique 37.5%
Fracture gap at time of presentation in mm	1.8 mm (range: 0.6–2.9)		
Humpback deformity Average lateral angle	75%; 68° (range: 22.7–107°)		
Dorsal intercalated segment instability Average angle	75%; 23.83° (range: 18–36°)		

**Table 2:** Bone consolidation 6 weeks after surgery.

	Distal Interface			Proximal Interface		
	MDCT		HR-pQCT	MDCT		HR-pQCT
Healed (>75%)	87.5%	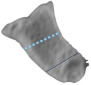	37.5%	12.5%	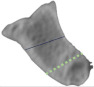	25%
>50% bone consolidation	12.5%	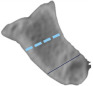	62.5%	62.5%	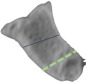	75%
<50% bone consolidation	0%	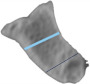	0%	25%	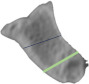	0%
Non-union	0%	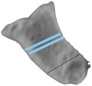	0%	0%	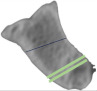	0%

**Table 3:** Bone consolidation 12 weeks after surgery.

	Distal Interface			Proximal Interface		
	MDCT		HR-pQCT	MDCT		HR-pQCT
Healed (>75%)	87.5%	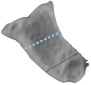	75%	37.5%	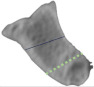	50%
>50% bone consolidation	12.5%	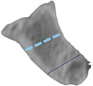	25%	62.5%	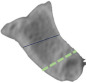	50%
<50% bone consolidation	0%	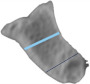	0%	0%	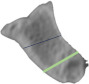	0%
Non-union	0%	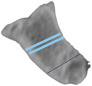	0%	0%	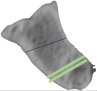	0%

**Table 4:** Thumb strength in kg at time of surgery and during follow-up period.

	Baseline	6 Weeks Post-Op	3 Months Post-Op	6 Months Post-Op	12 Months Post-Op	5–6 Years Post-Op
Affected side: *	Ø 6.7	Ø 2.5	Ø 6.3	Ø 4	Ø 5.5	Ø 10
Contralateral side: *	Ø 7.2	Ø 5	Ø 9.5	Ø 6.6	Ø 6.6	Ø 11.5

* mean in kg.

**Table 5:** Grip strength in kg at time of surgery and during follow-up period.

	Baseline	3 Months Post-Op	6 Months Post-Op	12 Months Post-Op	24 Months Post-Op	5–6 Years Post-Op
Affected side: *	Ø 34	Ø 21	Ø 41	Ø 45	Ø 51	Ø 43
Contralateral side: *	Ø 44	Ø 40	Ø 50	Ø 50	Ø 56	Ø 46

* mean in kg.

**Table 6:** DASH scores of all patients at time of surgery and during follow-up period.

Baseline	6 Weeks Post-Op	3 Months Post-Op	6 Months Post-Op	12 Months Post-Op	24 Months Post-Op	5–6 Years Post-Op
49.17	40.83	26.67	22.5	10.83	7.5	4
71.67	63.33	46.67	40	25.83	17.5	15
76.67	63.33	58.33	51.67	21.67	15	15
3.33	30.83	14.17	10	4	0	0
60.00	70.83	55	17.5	6.67	3	2
43.33	46.67	21.67	15	5	2	1
20.00	45	14.17	9	4	0	0
11.67	40.83	48.33	30	12	0	0

**Table 7:** PRWE scores of all patients at time of surgery and at follow-up.

Baseline	6 Weeks Post-Op	3 Months Post-Op	6 Months Post-Op	12 Months Post-Op	24 Months Post-Op	5–6 Years Post-Op
56	51	27	28	17	13	9
82	80	64	67	65	50	43
45	36	53	47	31	22	15
15	37	18	16	14	11	9
40	42	35	24	18	17	18
41	40	23	21	16	10	6
20	55	40	36	31	31	25
41	31	65	61	60	51	38

## Data Availability

The original contributions presented in this study are included in the article. Further inquiries can be directed to the corresponding authors.

## References

[1] Schmitt R., Rosenthal H. (2016). Imaging of Scaphoid Fractures According to the New S3 Guidelines. RoFo: Fortschritte auf dem Gebiete der Rontgenstrahlen und der Nuklearmedizin.

[2] Neubrech F., Terzis A., Seegmüller J., Sauerbier M. (2019). Diagnostics and treatment of acute scaphoid fractures. Unfallchirurg.

[3] Janowski J., Coady C., Catalano L.W. (2016). Scaphoid Fractures: Nonunion and Malunion. J. Hand Surg..

[4] Merrell G.A., Wolfe S.W., Slade J.F. (2002). Treatment of scaphoid nonunions: Quantitative meta-analysis of the literature. J. Hand Surg..

[5] Robbins R.R., Ridge O., Carter P.R. (1995). Iliac crest bone grafting and Herbert screw fixation of nonunions of the scaphoid with avascular proximal poles. J. Hand Surg..

[6] Daly K., Gill P., Magnussen P.A., Simonis R.B. (1996). Established nonunion of the scaphoid treated by volar wedge grafting and Herbert screw fixation. J. Bone Jt. Surg. Br. Vol..

[7] Smith B.S., Cooney W.P. (1996). Revision of failed bone grafting for nonunion of the scaphoid. Treatment options and results. Clin. Orthop. Relat. Res..

[8] Chang M.A., Bishop A.T., Moran S.L., Shin A.Y. (2006). The outcomes and complications of 1,2-intercompartmental supraretinacular artery pedicled vascularized bone grafting of scaphoid nonunions. J. Hand Surg..

[9] Steinmann S.P., Adams J.E. (2006). Scaphoid fractures and nonunions: Diagnosis and treatment. J. Orthop. Sci. Off. J. Jpn. Orthop. Assoc..

[10] Tsumura T., Matsumoto T., Matsushita M., Ono K., Kishimoto K., Shiode H. (2020). How Long Should We Immobilize the Wrist after Vascularized Bone Grafting for the Treatment of Scaphoid Nonunion?. J. Hand Surg. Asian Pac..

[11] Muirhead C., Talia A., Fraval A., Ross A., Thai D. (2021). Early mobilization vs delayed mobilisation following the use of a volar locking plate with non-vascularized bone graft in scaphoid non-union. A multicentred randomised controlled-trial. J. Orthop..

[12] Benedikt S., Stock K., Horling L., Schmidle G., Schirmer M., Degenhart G., Blauth M., Lamina C., Pallua J.D., Arora R. (2025). Bone remodelling after scaphoid fractures: HR-pQCT, clinical and laboratory data from a prospective 1-year follow-up study. Bone.

[13] Clementson M., Björkman A., Thomsen N.O.B. (2020). Acute scaphoid fractures: Guidelines for diagnosis and treatment. EFORT Open Rev..

[14] Fernandez D.L. (1984). A technique for anterior wedge-shaped grafts for scaphoid nonunions with carpal instability. J. Hand Surg..

[15] Burghardt A.J., Link T.M., Majumdar S. (2011). High-resolution computed tomography for clinical imaging of bone microarchitecture. Clin. Orthop. Relat. Res..

[16] Burghardt A.J., Kazakia G.J., Link T.M., Majumdar S. (2009). Automated simulation of areal bone mineral density assessment in the distal radius from high-resolution peripheral quantitative computed tomography. Osteoporos. Int. A J. Establ. Result Coop. Eur. Found. Osteoporos. Natl. Osteoporos. Found. USA.

[17] Whittier D.E., Boyd S.K., Burghardt A.J., Paccou J., Ghasem-Zadeh A., Chapurlat R., Engelke K., Bouxsein M.L. (2020). Guidelines for the assessment of bone density and microarchitecture in vivo using high-resolution peripheral quantitative computed tomography. Osteoporos. Int. A J. Establ. Result Coop. Eur. Found. Osteoporos. Natl. Osteoporos. Found. USA.

[18] Daniels A.M., Bevers M., Sassen S., Wyers C.E., van Rietbergen B., Geusens P., Kaarsemaker S., Hannemann P.F.W., Poeze M., van den Bergh J.P. (2020). Improved Detection of Scaphoid Fractures with High-Resolution Peripheral Quantitative CT Compared with Conventional CT. J. Bone Jt. Surg. Am. Vol..

[19] Daniels A.M., Wyers C.E., Janzing H.M.J., Sassen S., Loeffen D., Kaarsemaker S., van Rietbergen B., Hannemann P.F.W., Poeze M., van den Bergh J.P. (2020). The interobserver reliability of the diagnosis and classification of scaphoid fractures using high-resolution peripheral quantitative CT. Bone Jt. J..

[20] Bevers M., Daniels A.M., Wyers C.E., van Rietbergen B., Geusens P., Kaarsemaker S., Janzing H.M.J., Hannemann P.F.W., Poeze M., van den Bergh J.P.W. (2020). The Feasibility of High-Resolution Peripheral Quantitative Computed Tomography (HR-pQCT) in Patients with Suspected Scaphoid Fractures. J. Clin. Densitom. Off. J. Int. Soc. Clin. Densitom..

[21] de Jong J.J., Willems P.C., Arts J.J., Bours S.G., Brink P.R., van Geel T.A. (2014). Assessment of the healing process in distal radius fractures by high resolution peripheral quantitative computed tomography. Bone.

[22] Bevers M.S.A.M., Daniels A.M., van Rietbergen B., Geusens P.P.M.M., van Kuijk S.M.J., Sassen S., Kaarsemaker S., Hannemann P.F.W., Poeze M., Janzing H.M.J. (2021). Assessment of the healing of conservatively-treated scaphoid fractures using HR-pQCT. Bone.

[23] Nishino Y., Chiba K., Era M., Okazaki N., Miyamoto T., Yonekura A., Tomita M., Osaki M. (2020). Analysis of fracture healing process by HR-pQCT in patients with distal radius fracture. J. Bone Miner. Metab..

[24] Era M., Chiba K., Nishino Y., Okazaki N., Miyamoto T., Yonekura A., Tomita M., Tsurumoto T., Osaki M. (2019). The effects of volar locking plates for distal radius fractures on the image quality of high-resolution peripheral quantitative computed tomography. Bone.

[25] Ramamurthy C., Cutler L., Nuttall D., Simison A.J., Trail I.A., Stanley J.K. (2007). The factors affecting outcome after non-vascular bone grafting and internal fixation for nonunion of the scaphoid. J. Bone Jt. Surg. Br. Vol..

[26] MacDermid J.C., Turgeon T., Richards R.S., Beadle M., Roth J.H. (1998). Patient rating of wrist pain and disability: A reliable and valid measurement tool. J. Orthop. Trauma.

[27] Beaton D.E., Katz J.N., Fossel A.H., Wright J.G., Tarasuk V., Bombardier C. (2001). Measuring the whole or the parts? Validity, reliability, and responsiveness of the Disabilities of the Arm, Shoulder and Hand outcome measure in different regions of the upper extremity. J. Hand Ther. Off. J. Am. Soc. Hand Ther..

[28] Singh H.P., Forward D., Davis T.R., Dawson J.S., Oni J.A., Downing N.D. (2005). Partial union of acute scaphoid fractures. J. Hand Surg..

[29] Grewal R., Frakash U., Osman S., McMurtry R.Y. (2013). A quantitative definition of scaphoid union: Determining the inter-rater reliability of two techniques. J. Orthop. Surg. Res..

[30] de Jong J.J., Arts J.J., Meyer U., Willems P.C., Geusens P.P., van den Bergh J.P., van Rietbergen B. (2016). Effect of a Cast on Short-Term Reproducibility and Bone Parameters Obtained from HR-pQCT Measurements at the Distal End of the Radius. J. Bone Jt. Surg. Am. Vol..

[31] Peters M., Scharmga A., van Tubergen A., Arts J., Loeffen D., Weijers R., van Rietbergen B., Geusens P., van den Bergh J.P. (2017). The Reliability of a Semi-automated Algorithm for Detection of Cortical Interruptions in Finger Joints on High Resolution CT Compared to MicroCT. Calcif. Tissue Int..

[32] Krug R., Burghardt A.J., Majumdar S., Link T.M. (2010). High-resolution imaging techniques for the assessment of osteoporosis. Radiol. Clin. N. Am..

[33] Pialat J.B., Burghardt A.J., Sode M., Link T.M., Majumdar S. (2012). Visual grading of motion induced image degradation in high resolution peripheral computed tomography: Impact of image quality on measures of bone density and micro-architecture. Bone.

[34] Matsuki H., Ishikawa J., Iwasaki N., Uchiyama S., Minami A., Kato H. (2011). Non-vascularized bone graft with Herbert-type screw fixation for proximal pole scaphoid nonunion. J. Orthop. Sci. Off. J. Jpn. Orthop. Assoc..

[35] Munk B., Larsen C.F. (2004). Bone grafting the scaphoid nonunion: A systematic review of 147 publications including 5,246 cases of scaphoid nonunion. Acta Orthop. Scand..

[36] Amadio P.C., Berquist T.H., Smith D.K., Ilstrup D.M., Cooney W.P., Linscheid R.L. (1989). Scaphoid malunion. J. Hand Surg..

[37] GmbH A-ÖAfGuE (2019). Strahlenschutz—Unser Leistungsspektrum im Strahlenschutz.

[38] Guss M.S., Mitgang J.T., Sapienza A. (2018). Scaphoid Healing Required for Unrestricted Activity: A Biomechanical Cadaver Model. J. Hand Surg. Am..

